# GC–MS Analysis and In Vivo and Ex Vivo Antidiarrheal and Antispasmodic Effects of the Methanolic Extract of *Acacia nilotica*

**DOI:** 10.3390/molecules27072107

**Published:** 2022-03-24

**Authors:** Najeeb Ur Rehman, Mohd Nazam Ansari, Wasim Ahmad, Mohd Amir

**Affiliations:** 1 Department of Pharmacology & Toxicology, College of Pharmacy, Prince Sattam Bin Abdulaziz University, Al-Kharj 11942, Saudi Arabia; 2 Department of Pharmacy, Mohammed Al-Mana College for Medical Sciences, Dammam 34222, Saudi Arabia; wasimahmadansari@yahoo.com; 3 Department of Natural Products and Alternative Medicine, College of Clinical Pharmacy, Imam Abdulrahman Bin Faisal University, Dammam 31441, Saudi Arabia; matahmad@iau.edu.sa

**Keywords:** *A*. *nilotica*, antispasmodic, Ca^++^ channel blocker, GC-MC, phosphodiesterase inhibitor

## Abstract

This present study evaluated and rationalized the medicinal use of the fruit part of *Acacia nilotica* methanolic extract. The phytochemicals were detected using gas chromatography–mass spectrometry (GC–MS) while the in vivo antidiarrheal test was done using Swiss albino mice. To determine the details of the mechanism(s) involved in the antispasmodic effect, isolated rat ileum was chosen using different ex vivo assays by maintaining a physiological environment. GC–MS results showed that *A*. *nilotica* contained pyrogallol as the major polyphenol present (64.04%) in addition to polysaccharides, polyphenol, amino acid, steroids, fatty acid esters, and triterpenoids. In the antidiarrheal experiment, *A. nilotica* inhibited diarrheal episodes in mice significantly (*p* < 0.05) by 40% protection of mice at 200 mg/kg, while 80% protection was observed at 400 mg/kg by the orally administered extract. The highest antidiarrheal effect was observed with loperamide (*p* < 0.01), used as a control drug. In the ex vivo experiments, *A. nilotica* inhibited completely in increasing concentrations (0.3 to 10 mg/mL) the carbachol (CCh; 1 µM) and high K^+^ (80 mM)-evoked spasms in ileum tissues at equal potencies (*p* > 0.05), similar to papaverine, a dual inhibitor of the phosphodiesterase enzyme (PDE) and Ca^++^ channels. The dual inhibitory-like effects of *A. nilotica* on PDE and Ca^++^ were further validated when *A. nilotica* extract (1 and 3 mg/mL)-pre-incubated ileum tissues potentiated and shifted isoprenaline relaxation curves towards lower doses (leftward), similar to papaverine, thus confirming the PDE inhibitory-like mechanism whereas its CCB-like effect of the extract was confirmed at 3 and 5 mg/mL by non-specific inhibition of CaCl_2_-mediated concentration response curves towards the right with suppression of the maximum peaks, similar to verapamil, used as standard CCB. Thus, this study characterized the chemical composition and provides mechanistic support for medicinal use of *A. nilotica* in diarrheal and hyperactive gut motility disorders.

## 1. Introduction

Gastrointestinal (GI) motility plays an important role in digestive and absorptive processes of the gut, essential for pushing intestinal material, mixing this with digestive juices, and preparing undigested foods for excretion. Diarrhea, characterized by an increased frequency of bowel movements, wet stool, and abdominal cramps, is a serious health problem [1,2]. Diarrhea can be caused by several factors, such as infections, food intolerance, intestinal disorders, etc. [3,4,5], and might be a symptom of many other ailments, including IBS and diabetes [6,7]. Gut motility is controlled via various physiological agents, such as, acetylcholine (ACh), prostaglandin E2, serotonin (5-hydroxytryptamine or 5-HT), histamine, substance P, and cholecystokinins [8,9]. These chemicals cause excitatory actions that eventually increase cytosolic Ca^++^ [10]. Thus, any material which has the ability to interfere with the above specific pathways (PDE-inhibitory, adrenergic or opioid receptors activation) or with non-specific suppressant activities (Ca^++^ channel antagonists) is thought to be efficient in hypermotile gut conditions [9].

Currently available treatments for diarrhea are non-specific and generally, drugs are used to reduce the uneasiness and discomfort of recurrent bowel movements [11]. Available antidiarrheal drugs such as loperamide used to reduce motility may prevent diarrhea, and antispasmodic drugs diminish intestinal contraction and decrease pain [12,13]. Antimuscarinic and other antispasmodic drugs are a valuable therapy in diarrhea including IBS because the smooth muscle relaxant properties of these drugs reduce intestinal spasms [12,13]. Since time immemorial, plants have been used as a source to provide humankind with medicines having high therapeutic potential to treat health disorders and to combat numerous pathogenic infections [14]. The healing property of medicinal plants has been widely used in different traditional systems of medicine such as Ayurvedic, Unani, and Chinese [15,16]. This healing ability is attributed to the presence of various classes of compounds present in medicinal plants [17].

*Acacia nilotica* (L.) Wild ex. Del., commonly known as *Mimosa nilotica* (family Mimosaceae), is a medium-sized tree that is known locally as ‘Babul’ or ‘Kikar’ [18]. Africa, the Arabian Peninsula, and the Indian subcontinent have suitable environmental conditions for the growth of *A. nilotica* [19]. Other Acacia species, such as *A. arabica*, *A. abyssinica*, and *A. seyal*, are used in traditional medicine to treat leprosy, tuberculosis, skin ulcers, dysentery, cough, smallpox, toothache, and malignancies, as well as used as astringents, antispasmodics, antidysentrics, and aphrodisiacs [18,19,20]. Pods and tender leaves are used to treat diarrhea [21] and are also thought to be very effective in treating diabetes mellitus in folk medicine [22]. In recent studies, the plant has been reported for its intriguing bioactivities, such as antibacterial, hypolipidemic, and antidiabetic actions [23,24,25]. Phytochemical analysis revealed the presence of polyphenolic chemicals and flavonoids in the flowers, as well as glycosides, organic acids, carbohydrates, volatile oils, tannins, and coumarins in the fruits [26]. *A. nilotica* is a possible source of antioxidant polyphenols [27,28,29], and including these antioxidants in functional meals possibly might help in the prevention of certain diseases.

Although *A. nilotica*, the plant applied in this research, is used in local folk medicine to treat a range of ailments, there is no solid scientific data to support the use of *A. nilotica* fruit extract in the treatment of diarrhea. As a result, the aim of the work was to use in vivo and ex vivo experiments for phytochemical investigation using GC–MS as well as to discover the exact mechanism(s) implicated in the putative gastrointestinal inhibitory effects of *A. nilotica* fruit extract.

## 2. Materials and Methods

### 2.1. Extraction of Plant Material

After purchasing the fruit of *A. nilotica* from a local market in Dammam (Saudi Arabia), it was identified and authenticated using macroscopic and microscopical examination by Dr. Abuzer Ali, Department of Pharmacognosy, Taif University, Taif Saudi Arabia and matched with a Pharmacopoeial standard. (The Unani Pharmacopoeia Part I issue IV; 2009). The plant sample was preserved at the herbarium with the voucher # PL/0445/2020-21/P-008. With the use of a mixer grinder, the plant material was powdered; 40 g of powdered crude sample was placed in a Soxhlet device and extracted with 200 mL of methanol at 70 degrees Celsius. The extract was concentrated using a rotary evaporator after rigorous extraction (Buchi, R-215; Schaffhausen, Switzerland). For future investigation, the concentrated extract was maintained in an airtight glass jar at 5–10 °C. The extract was GC–MS examined using earlier reported methods [30,31].

### 2.2. Chemicals

Sigma provided carbamylcholine (CCh), papaverine, isoprenaline, Ethylenediaminetetraacetic Acid (EDTA), verapamil, acetylcholine perchlorate (ACh), and loperamide (St. Louis, MO, USA). To make the physiological buffer solution (Tyrode), the following reagents (salts) were used: magnesium sulphate, potassium chloride, glucose, potassium dihydrogen phosphate, calcium chloride, sodium chloride, and sodium bicarbonate (Merck, Darmstadt, Germany). All of the substances were of analytical quality, except castor oil acquired from a local drugstore.

### 2.3. Animals

From the Animal Care Unit, ‘College of Pharmacy, Prince Sattam bin Abdulaziz University, Saudi Arabia’, Swiss albino mice (25–30 g) were obtained for in vivo studies and rats (200–250 g) for ex vivo experiments and were kept at a temperature optimum (22 °C), relative humidity (55%), and exposure to a light/dark cycle. All animals were fed a regular diet of pellets and had unrestricted access to water. Prior to the ex vivo studies, mice fasted for 24 h, and cervical dislocation was performed under light sedation, with death confirmed by elimination of ear reflexes. All experiments (in vivo and ex vivo) were carried out with caution and in accordance with the guidelines outlined in the NRC [32]. The Bio-Ethical Research Committee (BERC) at ‘Prince Sattam Bin Abdulaziz University’ approved the study protocol with the approval number BERC-004-12-19.

### 2.4. GC–MS Analysis

The phytochemical investigation of the methanolic extract of *A. nilotica* was per-formed by GC–MS to detect the presence of several phytoconstituents. The chromatographic separation of metabolites was carried on a capillary column 60 M TRX 5-MS (30 m × 250 µm I.D. 0.25 µm film) using 2 μL of sample injection volume. The oven temperature program was as follows: 80 °C initially for 3 min and then ramped at a rate of 10 °C/min to 280 °C for 19 min. The carrier gas was set at a constant flow rate of 1.21 mL/min. The injection port, transfer line, and ion source were set to 260 °C, and the mass-scanning range was set to 40 to 650 *m*/*z* in scan mode. The injection was executed in split mode with a 10:1 split ratio, and a 3-min solvent delay time was set for the samples. Identification of individual phytoconstituents was achieved using National Institute of Standards and Technology (NIST) libraries and the mass spectra of literature [30,33].

### 2.5. In Vivo Antidiarrheal Study

Twenty mice were divided into five groups, each with an equal number of mice, at random. Mice in the first group were administered an oral gavage of saline (10 mL/kg) after a twenty-four-hour fast and were labeled as the negative control group. The second and third groups (test groups) were given two increasing doses of *A. nilotica* methanolic extract, 200 and 400 mg/kg, respectively, after a pilot screening for dose selection. As a positive control, the fourth group of mice was administered loperamide (10 mg/kg). Each animal was kept in the cage, with a blotting sheet on the floor to allow a blind observer to determine the presence or absence of diarrhea. All mice were given castor oil (10 mL/kg) orally after an hour. All blotting sheets from individual cages were checked for typical diarrheal droppings after 4 h. If no diarrheal spots were noticed on the blotting sheet, protection was documented [34,35].

### 2.6. Ex Vivo Experiments on Isolated Rat Ileum

A previously documented approach was used to sacrifice rats and to separate the final part of the small intestine (ileum) [36]. Ileum tissues (2–3 cm) were cleaned from neighboring tissues and luminal feces and mounted in an isolated organ bath (emkaBATH, Paris, France) attached to transducer and IOX software. The temperature was set to 37 °C, and a freshly prepared Tyrode’s solution bubbled with carbogen gas was provided as a physiological medium in the tissue baths (20 mL). The composition of Tyrode’s solution in mM was 2.68 KCl, 136.9 NaCl, 1.05 MgCl_2_, 11.90 NaHCO_3_, 0.42 NaH_2_PO_4_, 1.8 CaCl_2_, and 5.55 glucose, (pH 7.4) The tissues were stabilized for 30 min with the addition of acetylcholine (0.3 M) at regular intervals (5 min) while 1 g tension was applied by clockwise rotation of the transducer knob. CCh and high K^+^ (80 mM) were employed to induce prolonged contractions after stabilization, and *A. nilotica* was added to the bath solution in increasing concentrations until the maximal and/or complete relaxation of tissue was achieved. The inhibitory effect of *A. nilotica* on CCh and K^+^-mediated contractions was observed, which could indicate pharmacodynamics such as voltage-gated Ca^++^ channel blockade and/or PDE inhibition. Multiple smooth muscles are depolarized by K^+^ (>30 mM), which activates Ca^++^ channels (L-type), resulting in prolonged contractions [37]. PDE-inhibitors, on the other hand, are agents that, at comparable concentrations, reverse CCh and high K^+^-mediated contractions, whereas verapamil (CCB) shows significantly higher potency against high K^+^ compared to CCh-mediated contractions [38].

### 2.7. Ca^++^ Inhibitory Confirmation

After the observation of preliminary relaxation of *A. nilotica* against high K^+^, ileum tissues were incubated in Ca^++^-free Tyrode’s solution with EDTA (0.1 mM) for 45 min to confirm Ca^++^ channel blocking (CCB). A Ca^++^-free solution was replaced with a K^+^-rich and Ca^++^-free Tyrode’s solution at the following concentrations (mM): KCl 50, NaCl 91.04, MgCl_2_ 1.05, NaHCO_3_ 11.90, NaH_2_PO_4_ 0.42, glucose 5.55, and EDTA 0.1. After 45 min of incubation in this solution in the presence and absence of increasing concentrations of *A. nilotica*, CaCl_2_ CRCs were produced, and the findings were compared to the standard CCB agent, verapamil [39].

### 2.8. PDE Inhibitory Confirmation

The relaxing effect of *A. nilotica* against high K^+^ and CCh at identical concentrations is an indication of PDE inhibition [40]; therefore, dose-mediated inhibitory curves of isoprenaline against CCh in the presence and absence of *A. nilotica* were used to indirectly validate PDE inhibition. PDE blockage was indicated by the potentiation of isoprenaline curves to the left, similar to papaverine, a typical PDE inhibitor, utilized as a control [41].

### 2.9. Statistical Analysis

The statistical analyses were performed as the mean ± standard error of the mean (SEM), with “*n*” being the number of experiments that were repeated. The median effective concentrations (EC_50_) are geometric means with 95% confidence intervals (CIs). The statistical criteria utilized for multiple comparisons of concentration–response curves (CRCs) with controls were Student’s t-test or two-way ANOVA followed by Bonferroni’s post-test. W the Chi-square (χ^2^) test, all groups were statistically compared to a saline control group for diarrhea protection. *p* < 0.05 was regarded as statistically significant. For CRC regression analysis, Graph Pad Prism (version 4) was used.

## 3. Results

### 3.1. Methanolic Extract Yield (%)

The fruits of *A. nilotica* yielded 36.47% of methanolic crude extract.

### 3.2. GC–MS Phytochemical Profiling

**Figure 1:**
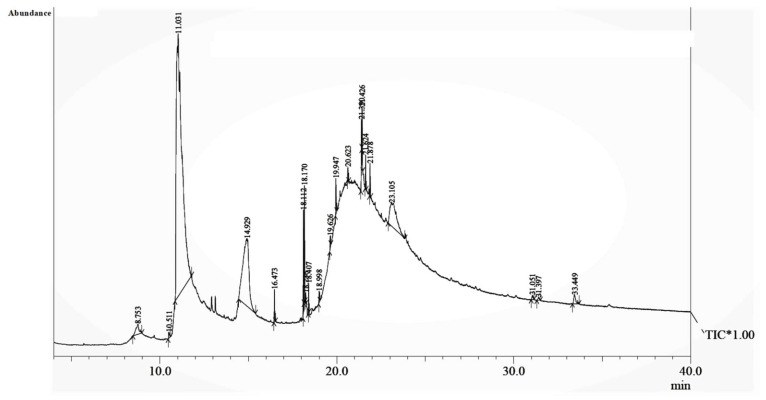
GC–MS chromatogram of methanolic extract of *A. nilotica*.

The phytochemical investigation of the *A. nilotica* methanolic extract revealed the presence of 19 phytoconstituents representing 99.03% that were identified by compairing with mass spectrum library of NIST. All separated phytoconstituents, peak area, % area, retention index, and molecular formula with the chemical structure of *A. nilotica* are shown in Table 1. Phytochemical investigation of methanolic extract showed the presence of polysaccharides, polyphenol, amino acid, steroids, and fatty acid esters. Pyrogallol (64.04%), 4-O methylmannose (17.7), 9,12-Octadecadienoic acid (6.8%), methyl oleate (1.9%), methyl linoleate (1.6%) and N,N-Dimethylglycine (1.3%) were the major phytoconstituents found in *A. nilotica*. These phytoconstituents were tentatively identified by compairing their mass spectra with the NIST library (Table 1 and Figure 1).

### 3.3. In Vivo Antidiarrheal Effect

In comparison to the saline group, both increasing orally delivered dosages of *A. nilotica* in mice showed significant antidiarrheal effects (Table 2). At the lower tested dose of 200 mg/kg, two out of five mice showed protection, suggesting 40% protection, whereas the higher dose of 400 mg/kg demonstrated 80% protection from diarrhea. In all five cages of mice treated with loperamide (10 mg/kg), no diarrheal spot was observed (100% protection), as detailed in Table 2.

### 3.4. Ex Vivo Antispasmodic Effects

**Figure 2:**
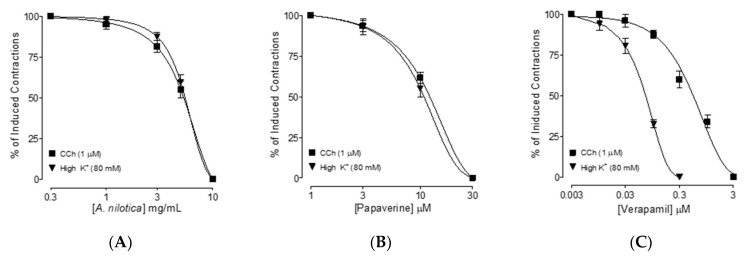
Concentration–response curves showing comparison of the (**A**) methanolic extract of *A. nilotica*, (**B**) papaverine, and (**C**) verapamil, for the inhibitory effect against carbachol (CCh; 1 µM) and high K^+^ (80 mM)-induced contractions in isolated rat ileum preparations. Values shown are the mean ± SEM, *n* = 4–5.

As demonstrated in Figure 2A, *A. nilotica* completely inhibited CCh and high K^+^-mediated spasm in rat isolated ileal tissues, with EC_50_ values of 5.48 mg/mL (4.85–6.26, 95 percent CI, *n* = 4–5) and 5.84 mg/mL (4.28–6.64, 95 percent CI, *n* = 4–5), respectively. Papaverine had similar relaxing effects on CCh and high K^+^-induced spasms, with EC_50_ values of 9.82 M (8.46–10.22, 95 percent CI, *n* = 4–5) and 9.24 M (7.98–10.92, 95 percent CI, *n* = 4–5), respectively (Figure 2B). As demonstrated in Figure 2C, verapamil had a much higher potency to block high K^+^ than CCh-evoked spasms, with EC_50_ values of 0.14 M (0.12–0.19, 95 percent CI, *n* = 4–5) and 2.82 M (2.44–2.94, 95 percent CI, *n* = 4–5), respectively.

### 3.5. Phosphodiesterase Enzyme (PDE)-Inhibitory like Effect

**Figure 3:**
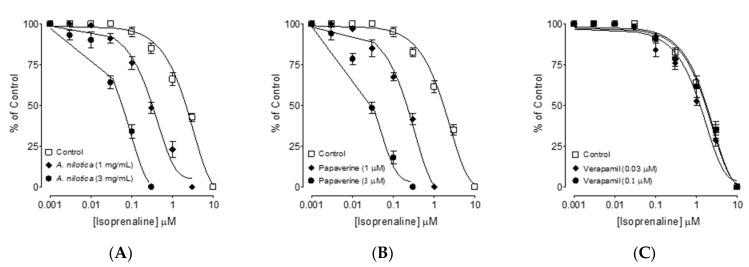
Inhibitory concentration–response curves of isoprenaline against carbachol (CCh)-induced contractions in the absence and presence of different concentrations of (**A**) the methanolic extract of *A. nilotica*, (**B**) papaverine, and (**C**) verapamil in isolated rat ileum preparations. Values shown are the mean ± SEM, *n* = 4–5.

Pretreatment with *A. nilotica* (1 and 3 mg/mL) confirmed PDE inhibitory activity by shifting the isoprenaline-induced inhibitory CRCs to the left (Figure 3A), indicating a potentiating impact. Papaverine (1 and 3 µM) generated a comparable leftward shift in the isoprenaline curves, as seen in Figure 3B, while verapamil had no potentiating impact (Figure 3C).

### 3.6. Calcium Channel Blocking (CCB)-like Effect

**Figure 4:**
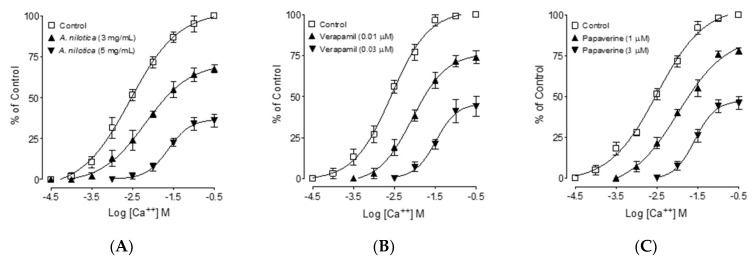
Concentration–response curves of Ca^++^ in the absence and presence of the increasing concentrations of the (**A**) methanolic extract of *A. nilotica*, (**B**) verapamil, and (**C**) papaverine in isolated rat ileum preparations. Values shown are the mean ± SEM, *n* = 4–5.

To confirm the Ca^++^ inhibitory activity, preincubation of ileum tissues with *A. nilotica* methanolic extract skewed the Ca^++^ CRCs curves at tested dosages of 3 and 5 mg/mL (Figure 4A) towards the right with suppression of the maximum effect. Similarly, verapamil and papaverine, at respective preincubated concentrations (0.01 and 0.03 µM; verapamil) and (1 and 3 µM; papaverine), also deflected Ca^++^ curves towards the right with suppression of the highest peaks as shown in Figure 4B,C.

## 4. Discussion

To provide the basis to the traditional medicinal report of *A. nilotica* in diarrhea and gut spasms [42], the methanolic extract of *A. nilotica* was evaluated scientifically using rodents while its phytochemical analysis was determined by GC–MS. In vivo, *A. nilotica* was examined in a castor oil-evoked diarrhea model and found to have dose-mediated antidiarrheal action by preventing the characteristic diarrheal drops as compared to the saline control group. Castor oil is known to increase intestinal fluids, causing diarrhea indirectly through the formation of recinoleic acid, which ultimately alters the electrolytes and water transport and elicits excitations in transverse and distal segments of the colon [43]. Similar to the positive control drug, loperamide, a popular antidiarrheal treatment [44], pre-administration of *A. nilotica* protected mice from diarrhea in a dose-dependent manner. The methanolic extract of *A. nilotica* was examined at cumulative doses in isolated rat ileum to determine the possible pharmacodynamics involved in the observed antidiarrheal activity [45]. Based on earlier results that antispasmodic drugs mediate gut inhibitory effects via Ca^++^ channel blocking [44] and/or PDE inhibition [46], we evaluated *A. nilotica* extract on the evoked contractions in rat ileum by CCh and high K^+^ [47]. The EC_50_ values obtained from both types of inhibitory curves of *A. nilotica* against CCh and high K^+^ showed no statistical difference (*p* > 0.05). Similarly, papaverine, a dual Ca^++^ channel and PDE inhibitor [38], suppressed both CCh and high K^+^-evoked spasms at comparable concentrations, but verapamil, a typical CCB [39,48], selectively inhibited high K^+^ at a lower concentration compared to CCh. This indicates that, similar to papaverine, *A. nilotica* has dual inhibitory mechanisms for PDE inhibition and Ca^++^ channels. PDE-inhibitors, which block PDE, result in a cAMP increase in tissues and thus cause relaxation. PDE hinders smooth muscle relaxation by converting cAMP into its inactive form (AMP) [49]. Hence, *A. nilotica* was evaluated indirectly for PDE-inhibition and cAMP elevation by constructing isoprenaline-induced inhibitory CRCs in the absence and presence of pre-incubated tissues with the test substance. In pre-incubated ileum tissues of *A. nilotica*, potentiation of isoprenaline’s inhibitory CRCs towards lower dosages (leftward) verified its PDE-inhibitory character, and the results were equivalent to papaverine, a known PDE-inhibitor [50]. CCh-mediated smooth muscle spasm is well recognized to be inhibited by PDE inhibitors [51]. In order to explore the possibility of additional antispasmodic mechanisms in *A. nilotica* extract, it was tested for Ca^++^ ion inhibitory effect.

Substances that reverse high K^+^ (˃30 mM)-mediated spasm are considered as CCBs [52], hence to support and confirm further the CCB-like action of *A. nilotica*, in previously Ca^++^-free tissues, the ileum tissues were preincubated with *A. nilotica* at increasing concentrations. Ca^++^-CRCs were made in the absence of *A. nilotica* and pre-incubated tissues with *A. nilotica*, which repelled Ca^++^-CRCs to the right with suppression of the maximum peak, similar to papaverine, a dual inhibitor of PDE and Ca^++^ channels. The plant Ca^++^-CRC comparison with verapamil, a standard CCB [39], further confirmed the additional CCB-like mechanism of *A. nilotica*. Previously published findings of the antispasmodic effect of *A. nilotica* pods in rabbit jejunum support this CCB-like effect [42]. Polysaccharides, polyphenols, amino acids, steroids, fatty acid esters, and triterpenoids were found in the GC–MS analysis of the *A. nilotica* methanolic extract. Pyrogallol was discovered to be one of the major phytoconstituents of *A. nilotica*; it is a polyphenol that is present in (64.04%) the extract and has antibacterial activity [53], whereas 4-O methylmannose is present in the second highest concentration (17.72%); this is a polysaccharide that has been reported to have anti-alopecic, anti-cirrhotic, anti-neuropathic, cholesterolytic, lipotropic, and sweetening properties [54]. In COPD patients, N, N-dimethylglycine may be useful as a diagnostic of protein degradation.

## 5. Conclusions

These findings characterized the chemical composition of the methanolic extract of *A. nilotica* and indicates pyrogallol as the major polyphenol present in addition to the polysaccharide, polyphenol, amino acid, steroids, fatty acid esters, and triterpenoids. The in vivo antidiarrheal and ex vivo antispasmodic assays conducted in rodents indicate that *A. nilotica* possesses dose-mediated protection in mice from castor-oil induced diarrhea similar to loperamide while its preincubation in isolated rat ileum potentiated the isoprenaline-mediated inhibitory curves whereas the Ca^++^ CRCs were shifted towards right with suppression of the maximum response, thus confirming its antispasmodic effect possibly mediated by a combination of PDE-inhibition and Ca^++^ channels antagonist-like mechanisms, though additional mechanism(s) cannot be ignored.

## Figures and Tables

**Table 1:** List of Phytoconstituents present in *A. nilotica* methanolic extract.

S. No.	Compound Name	% Area	Retention Index	MoleculaR Weight	Molecular Formula	Chemical Structure	Cas No	Nature of Compound
1	N,N-Dimethylglycine	1.3	824	103	C_4_H_9_NO_2_	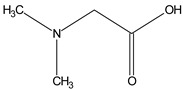	1118-68-9	Amino acid
2	4-methylbenzenethiol	0.2	1082	124.21	C_7_H_8_S	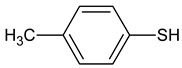	106-45-6	Thiol
3	Pyrogallol	64.0	1329	126.11	C_6_H_6_O_3_	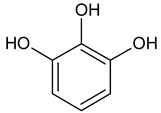	87-66-1	Polyphenol
4	1,8,11-Heptadecatriene, (Z,Z)-	0.6	1655	234.5	C_17_H_30_	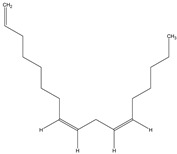	56134-03-3	Fatty Acid
5	4-O methylmannose	17.7	1714	194.18	C_7_H_14_O_6_	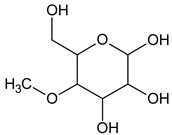	27552-11-0	Polysaccharide
6	Hexadecanoic acid, methyl ester	0.6	1905	270.5	C_17_H_34_O_2_	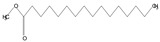	112-39-0	Fatty Acid ester
7	14,17-Octadecadienoic acid, methyl ester	0.1	2075	294.5	C_19_H_34_O_2_	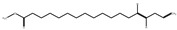	56554-60-0	Fatty Acid ester
8	9,12-Octadecadienoic acid (Z,Z)-	6.8	2078	280.4	C_18_H_32_O_2_	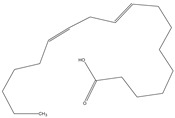	60-33-3	Fatty Acid
9	Methyl oleate	1.9	2081	296.5	C_19_H_36_O_2_	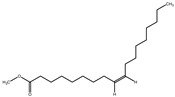	112-62-9	Fatty Acid ester
10	Methyl linoleate	1.6	2092	294.5	C_19_H_34_O_2_	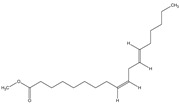	112-63-0	Fatty Acid ester
11	Methyl 9-cis,11-trans-octadecadienoate	0.2	2093	294.5	C_19_H_34_O_2_	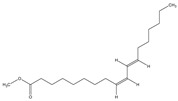	13058-52-1	Fatty Acid
12	Methyl stearate	0.4	2099	298.5	C_19_H_38_O_2_	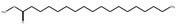	112-61-8	Fatty Acid
13	15-Hydroxypentadecanoic acid	0.5	2111	258.4	C_15_H_30_O_3_	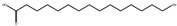	4617-33-8	Fatty Acid
14	Glycedyl palmitate	0.6	2241	312.5	C_19_H_36_O_3_	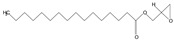	7501-44-2	Fatty Acid ester
15	Oxiranyl methyl ester 9-octadecenoic acid	0.7	2343	338.5	C_21_H_38_O_3_	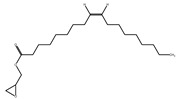	5431-33-4	Carboxylic ester
16	9-Octadecenamide	0.2	2375	281.5	C_18_H_35_NO	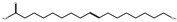	3322-62-1	Fatty Acid
17	Phthalic acid, bis(2-ethylhexyl) ester	0.5	2507	390.5	C_24_H_38_O_4_	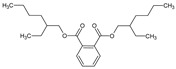	117-81-7	Carboxylic acid
18	Ergosta-5,22-dien-3-ol, (3.beta.,22E)-	0.2	3038	398.7	C_28_H_46_O	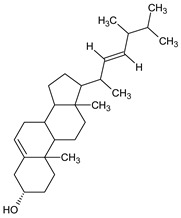	474-67-9	Steroid
19	Ergost-5-en-3-ol	0.1	3099	400.7	C_28_H_48_O	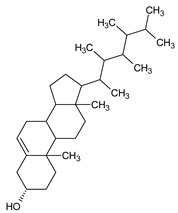	474-62-4	Steroid

**Table 2:** Antidiarrheal activity of the methanolic extract of *A. nilotica* on castor oil (10 mL/kg)-induced diarrhea in mice.

Treatment (p.o.), Dose (mg/kg)	No. of Mice with Diarrhea	% Protection
Saline (10 mL/kg) + Castor oil	5/5	0
*A. nilotica* + Castor oil		
200 (mg/kg) + 10 (mL/kg)	3 */5	40
400 (mg/kg) + 10 (mL/kg)	1 */5	80
Loperamide (10 mg/kg) + Castor oil	0 **/5	100

* *p* < 0.05 and ** *p* < 0.01 vs. Saline + Castor oil treated group (χ^2^-test).

## Data Availability

Not applicable.

## Sample Availability

Samples of the compounds are available from the authors.
